# Clinical overview and treatment options for non-skeletal manifestations of mucopolysaccharidosis type IVA

**DOI:** 10.1007/s10545-012-9459-0

**Published:** 2012-02-23

**Authors:** Christian J. Hendriksz, Maisoon Al-Jawad, Kenneth I. Berger, Sara M. Hawley, Rebecca Lawrence, Ciarán Mc Ardle, C. Gail Summers, Elizabeth Wright, Elizabeth Braunlin

**Affiliations:** 1 Birmingham Children’s Hospital NHS Foundation Trust, Birmingham, UK; 2 Institute of Dentistry, Queen Mary University London, London, UK; 3 André Cournand Pulmonary Physiology Laboratory, Bellevue Hospital, School of Medicine, New York University, New York, NY USA; 4 BioMarin Pharmaceutical Inc., Novato, CA USA; 5 Department of Ophthalmology, University of Minnesota, Minneapolis, MN USA; 6 Department of Pediatrics, University of Minnesota, Minneapolis, MN USA; 7 Clinical Inherited Metabolic Disorders, Birmingham Children’s Hospital NHS Foundation Trust, Steelhouse lane, Birmingham, B4 6NH UK

## Abstract

Mucopolysaccharidosis type IVA (MPS IVA) or Morquio syndrome is a multisystem disorder caused by galactosamine-6-sulfatase deficiency. Skeletal manifestations, including short stature, skeletal dysplasia, cervical instability, and joint destruction, are known to be associated with this condition. Due to the severity of these skeletal manifestations, the non-skeletal manifestations are frequently overlooked despite their significant contribution to disease progression and impact on quality of life. This review provides detailed information regarding the non-skeletal manifestations and suggests long-term assessment guidelines. The visual, auditory, digestive, cardiovascular, and respiratory systems are addressed and overall quality of life as measured by endurance and other functional abilities is discussed. Impairments such as corneal clouding, astigmatism, glaucoma, hearing loss, hernias, hepatomegaly, dental abnormalities, cardiac valve thickening and regurgitation, obstructive sleep apnea, tracheomalacia, restrictive and obstructive respiratory compromise, and muscular weakness are discussed. Increased awareness of these non-skeletal features is needed to improve patient care.

**Electronic supplementary material:**

The online version of this article (doi:10.1007/s10545-012-9459-0) contains supplementary material, which is available to authorized users.

## Introduction

MPS IVA (OMIM #253000), also known as Morquio syndrome, is an autosomal recessive lysosomal storage disease first described in 1929 (Morquio 1929; Brailsford 1929). Morquio syndrome can refer to either MPS IVA or MPS IVB, which, while similar, are caused by defects in different enzymes. MPS IVA is the focus of this review. The incidence of MPS IVA differs among different populations; reported rates range from 1 in 76,000 live births in Northern Ireland (Nelson 1997) to 1 in 640,000 live births in western Australia (Nelson et al. 2003).

MPS IVA is caused by mutation of the gene encoding galactosamine-6-sulfatase (GALNS, EC 3.1.6.4), which results in impaired catabolism of two glycosaminoglycans (GAGs), keratan sulfate and chondroitin-6-sulfate (Dorfman et al. 1976; Glössl and Kresse 1982). As in other MPS disorders, nondegraded GAGs accumulate in lysosomes to a degree that interferes with cellular function. The tissue distribution of the particular GAGs accumulated in an MPS disorder is reflected in the clinical presentation of the disease. In the case of MPS IVA, accumulation of keratan sulfate and chondroitin-6-sulfate manifests mainly as short stature and skeletal dysplasia (Wraith 1995) with bone deformity being the most common initial symptom (Montaño et al. 2007). Additional compromised systems include the visual, auditory, digestive, cardiovascular, and respiratory systems (Northover et al. 1996). The brain and spinal cord are not believed to be directly impacted by GAG accumulation in MPS IVA as normal intelligence is preserved (Wraith 1995). However, patients have a high risk of developing neurological complications due to skeletal abnormalities (Nelson and Thomas 1988).

There is a wide spectrum of disease progression among MPS IVA–affected individuals. A high degree of genetic heterogeneity is likely responsible for this phenotypic variety. More than 100 different mutations have been identified in the *GALNS* gene (Tomatsu et al. 2005). Clinical presentations of the disease range from a severe, rapidly progressing phenotype to a slowly progressing phenotype. Onset of disease symptoms commonly occurs prior to 1 year of age in rapidly progressing patients or as late as the second decade of life in slowly progressing patients (Montaño et al. 2007). Diagnosis is typically based on clinical examination, skeletal radiographs, urinary GAG tests, and the enzymatic activity of GALNS in blood cells or fibroblasts (Montaño et al. 2007). Further confirmation can be provided by identification of the molecular defect in the *GALNS* gene.

Once diagnosed, MPS IVA requires a multi-disciplinary approach to patient care. Management of skeletal manifestations and the associated neurological complications is critically important for patients with MPS IVA. However, the other aspects of the disease also significantly impact patient lives. MPS IVA–induced dysfunctions in the visual, auditory, digestive, cardiovascular, and respiratory systems can severely affect patient quality of life by reducing endurance, limiting participation in various activities, and increasing dependence on care givers. With the goal of establishing a better understanding of the non-skeletal manifestations of MPS IVA and improving patient quality of life, we systematically discuss the non-skeletal systems affected by MPS IVA, present recommended assessments, and discuss interventions and patient outcomes.

## Clinical features

### Visual system

Diffuse corneal clouding is the most common ocular finding in MPS IVA, although it occurs to a lesser extent and is more slowly progressive than in other MPS diseases (Danes 1973). A retrospective review of 20 patients with MPS IV (subtype A or B not specified), ages 1–65 years, identified 10 eyes with no corneal clouding, 17 eyes with mild corneal clouding, 4 with moderate corneal clouding, and 4 with severe corneal clouding (corneal clouding was not graded in 5 eyes); the severity of corneal clouding was generally related to increasing age of the patient and resulted in reduction in visual acuity (Couprie et al. 2010). The diffuse, finely granular corneal deposits are best visualized with slit-lamp biomicroscopy but when more severe, can be observed with a bright penlight held at an oblique axis to the eye. The corneal deposits may also interfere with examination of other ocular structures, e.g., the trabecular meshwork, the retina, and the optic nerve. Electron microscopy has shown that the corneal clouding is primarily due to fibrillogranular, as well as multimembranous, membrane-bound inclusions in the keratocytes, representing GAGs and complex lipids/glycolipids, respectively; in addition, extracellular granular material has been reported (Ghosh and McCulloch 1974; Iwamoto et al. 1990). This disruption of the normal corneal lamellae results in light scattering and can cause the patient to be photosensitive (Leslie et al. 2005). While the inclusions can also be found in the conjunctiva, sclera, trabecular meshwork, and retinal pigment epithelium, the primary effect is in the cornea (Iwamoto et al. 1990).

Glaucoma or ocular hypertension seems to be unusual in MPS IVA (Davis and Currier 1934). Case reports of corneal clouding and glaucoma in 35-year-old and 36-year-old siblings with MPS IV (subtype A or B not specified) found that the glaucoma was open angle (likely due to accumulation of GAG in the trabecular meshwork over time, obstructing aqueous outflow), was associated with visual field constriction, and was managed with topical medications (Cahane et al. 1990). This is in contrast to the closed/narrow angle glaucoma that has been reported in MPS VI (Cantor et al. 1989; Sato et al. 2002). Electron microscopy has shown distended trabecular endothelial cells with a thickened basement membrane in MPS IVA. The inclusions in the trabecular meshwork are primarily multimembranous, whereas other types of MPS have more fibrillogranular inclusions, perhaps explaining the reduced prevalence of ocular hypertension and glaucoma in individuals with MSP IVA (Iwamoto et al. 1990; Leslie et al. 2005).

While visual acuity is generally better in MPS IVA compared with other types of MPS, corneal clouding, refractive errors, glaucoma, and cataracts can affect visual acuity as the patient matures. Patients with MPS IV (subtype A or B not specified) tend to have astigmatism in addition to myopia and hyperopia (Couprie et al. 2010), in contrast with hyperopia alone as has been reported in patients with other types of MPS (Fahnehjelm et al. 2010). Different types of cataracts or lens opacities have been reported in MPS IV (subtype A or B not specified): punctate lens opacities in six individuals ages 6–45 years, “small” lens opacities in a 9-year-old, nuclear sclerosis in a 65-year-old, and lamellar/zonular cataracts in three individuals ages 8–15 years (Couprie et al. 2010; Iwamoto et al. 1990; Olsen et al. 1993). With progression of the cataracts, visual acuity may be affected.

Optic nerve atrophy may rarely occur due to glaucoma, optic nerve infiltration, or retinal dystrophy (Dangel and Tsou 1985; Abraham et al. 1974; Käsmann-Kellner et al. 1999). When retinal dystrophy is present, the patient may note nyctalopia (night blindness), and funduscopic examination can show constricted arterioles (Abraham et al. 1974; Dangel and Tsou 1985; Käsmann-Kellner et al. 1999). Electroretinography is needed to assess retinal function and make a diagnosis of a retinal dystrophy, typically reported as a reduction in the scotopic responses, in addition to possible delay in photopic implicit times (Dangel and Tsou 1985; Käsmann-Kellner et al. 1999; Abraham et al. 1974). Electroretinography has rarely been documented in MPS IVA, perhaps related to infrequent electrophysiology, absent or mild symptoms, or to the relative sparing of the retina and central nervous system as compared to other MPS disorders. One study, reporting reduced and delayed b-wave scotopic responses in a 43-year-old with MPS IV (subtype A or B not specified), noted that the retinal changes in these patients are insidious and might not be seen in younger patients (Dangel and Tsou 1985). Indeed, essentially normal retina and central nervous system tissue has been reported in MPS IVA with histopathology (Koto et al. 1978), and some clinical reports have documented normal electroretinography into the fourth decade of life (Cahane et al. 1990; Gills et al. 1965; Leung et al. 1971). Interestingly, no inclusions in retinal neurons and rare fibrillogranular inclusions in the retinal pigment epithelial cells were reported by one study (Iwamoto et al. 1990). The two cases with MPS IVA in this study, ages 18 and 19 years, also showed no optic nerve abnormalities on post-mortem examination (Iwamoto et al. 1990). Lastly, similar to other types of MPS, the eyes may appear prominent (pseudoexopthalmos) in MPS IVA due to shallow orbits (Käsmann-Kellner et al. 1999).

### Auditory system

As in all forms of MPS, reduction in hearing in patients with MPS IVA can be attributed to multiple causes. Firstly, conductive hearing loss can be present and is most likely secondary to recurrent upper respiratory tract infections and frequent serous otitis media (Schlieier and Steubel 1976). Conductive loss can also be caused by deformity of the ossicles (Barranger and Cabrera-Salazar 2002). Secondly, sensorineural loss may occur as a result of GAG accumulation. Abnormal auditory brainstem response (ABR) results have been described and are thought to be a combination of middle ear, cochlear, eighth nerve, and lower brainstem pathology (Leroy and Crocker 1966).

Hearing loss in MPS IVA usually begins in adolescence, however the conductive element caused by frequent upper respiratory tract infections and serous otitis media can be present anytime from birth onwards. The hearing loss is progressive, and once the sensorineural element is present, it can be severe and is almost universally found in patients who survive beyond the second decade (Bredenkamp et al. 1992; Keleman 1977).

Most patients with MPS IVA have a “mixed hearing loss” attributed to the combination of a conductive element and a sensorineural element. Ventilation tubes can be used to treat aspects of the conductive hearing loss, but the patient is likely to still have a conductive loss if there is ossicular involvement and most patients will have an underlying progressive sensorineural loss.

Abnormal vestibular function has also been reported in a patient with MPS IV (subtype A or B not specified) (Sataloff et al. 1987).

### Digestive system

The digestive system is also affected by MPS IVA. According to data from the International Morquio A Registry conducted by the International Morquio Organization, some MPS IVA patients have reported hernias among their initial and current symptoms (Tomatsu et al. 2011). Although umbilical and inguinal hernias are the most common hernia types in patients with MPS disorders (Ashworth et al. 2006), an MPS IV (subtype A or B not specified) patient with bilateral diaphragmatic hernias has also been reported (Nursal et al. 2000).

Another element of the digestive system that may be affected is the liver. Although not as common as in other MPS disorders, hepatomegaly has been reported in patients with MPS IVA (Nelson et al. 1988) and was reported as an initial and current symptom in the International Morquio A Registry (Tomatsu et al. 2011). Hepatosplenomegaly has also been reported (Holzgreve et al. 1981), although it seems to be less common and was not found in all studies in which it was evaluated (Nelson et al. 1988). The liver manifestations are likely a direct result of GAG accumulation as keratan sulfate and chondroitin-6-sulfate are known to accumulate in the liver (Minami et al. 1979). Interestingly, when analyzed, the keratan sulfate found in the liver of a patient with MPS IVA appeared to be structurally similar to the keratan sulfate found in bone (Minami et al. 1983).

In addition to hernias and hepatomegaly, generalized stomach problems were also reported by patients in the International Morquio A Registry (Tomatsu et al. 2011). While gastrointestinal dysfunction and chronic diarrhea have been reported in other MPS disorders (Wraith 1995; Sibilio et al. 2009), the specific stomach problems experienced by MPS IVA patients have not been described in the literature.

Overall, digestive system manifestations appear to be less prominent in MPS IVA than in other MPS disorders. However, there is one exception: dental abnormalities are a particular feature of MPS IVA (James et al. 2012). Due to the integral role of teeth in the digestive system and the importance of oral health in maintaining quality of life (Sheiham 2005), dental abnormalities are included here even though they could also be classified as a skeletal manifestation. Through in situ hybridization of a day 1 mouse incisor it has been shown that GALNS mRNA is most abundant in secretory ameloblasts indicative of a developmental disturbance during the secretory stage of enamel formation (Yamakoshi et al. 2002). This disturbance manifests as dental abnormalities in patients with MPS IVA including spaced dentition (Kuratani et al. 2005), pointed cusps (Rolling et al. 1999; Kinirons and Nelson 1990), spade-shaped incisors (Rolling et al. 1999; Kinirons and Nelson 1990), dental pitting with a band of increased porosity just below the surface of the enamel correlating to the positions of striae of Retzius (Rolling et al. 1999), enamel hypoplasia (Kuratani et al. 2005; Rolling et al. 1999; Kinirons and Nelson 1990), developmental abnormalities of primary and permanent dentition (James et al. 2012), and an increased risk of dental caries (James et al. 2012). In addition, a scanning electron microscopy study has revealed that MPS IV teeth possess a thin layer of amorphous material between the normal prismatic enamel and the dentine surface, believed to be the result of poorly mineralized tissue produced at the earliest stages of amelogenesis (Lustmann 1978). A recent study found that patients with MPS IVA have increased caries rates and enamel defects as compared to both the general population and patients with other MPS disorders (James et al 2012). In concurrence with this finding, there appears to be a marked difference in the orientation distribution of enamel crystallites between enamel affected by MPS IVA and healthy enamel (Al-Jawad et al. 2011).

### Cardiovascular system

Cardiac involvement in MPS IVA has previously been thought to be mild (Northover et al. 1996) or uncommon (Montaño et al. 2007). Accurate understanding of the cardiac findings in MPS IVA patients has been hampered by small patient numbers (Schieken et al. 1975; Gross et al. 1988), absence of supporting biochemical documentation of diagnosis (Schieken et al. 1975), failure to differentiate between MPS IVA and MPS IVB (Schieken et al. 1975; Gross et al. 1988; Wippermann et al. 1995; Dangel 1998; Mohan et al. 2002; Fesslová et al. 2009; Lael et al. 2010), and evolving cardiac ultrasound technology (Schieken et al. 1975; Gross et al. 1988; John et al. 1990; Dangel 1998). The single cardiac ultrasound study specifically addressing MPS IVA lacks the color flow Doppler technology that improves the detection of valve stenosis and insufficiency (John et al. 1990). Conversely, the studies that do utilize color Doppler technology do not differentiate between MPS IVA and MPS IVB (Schieken et al. 1975; Gross et al. 1988; Wippermann et al. 1995; Dangel 1998; Mohan et al. 2002; Fesslová et al. 2009; Lael et al. 2010).

Remarkably, despite the absence of color Doppler, cardiac valve disease was found to be quite common in the single study devoted to MPS IVA (John et al. 1990). Five of the 10 patients studied (50%) had mitral valve regurgitation (one of whom had mitral valve stenosis as well), 30% had aortic valve regurgitation, and 20% had both mitral and aortic regurgitation in conjunction with left ventricular hypertrophy. Aortic and/or mitral valve thickening was present in 40% of patients. The average age of patients within the study was 12.5 years although the range was broad (3–40 years). Valve dysfunction occurred in all three adults, but was also present in two patients aged 11 and 13 years. Studies that did not differentiate between MPS IVA and MPS IVB showed a comparable incidence of aortic and mitral valve thickening (40%) and valve stenosis (7–9%) but a somewhat lesser incidence of valve regurgitation (17–26%) (Supplemental Table 1).

It is generally thought that cardiac valves most affected in the MPS syndromes are those associated with dermatan sulfate deposition (Dangel 1998). However, keratan sulfate and chondroitin-6-sulfate are both found within normal cardiac valves (Latif et al. 2005), so it is not surprising that the gross and histological appearances of the MPS IVA cardiac valves are similar to the patterns found in MPS I, II, and VI. Valve thickening, seen on cardiac ultrasound, has been found to be nearly as common in patients who do not accumulate dermatan sulfate as in those who do (Lael et al. 2010). All MPS IVA cardiac valves may show GAG deposition, although the left-sided cardiac valves are more severely affected. Excess GAG is present within MPS IV (subtype A or B not specified) cardiac valve tissue obtained during valve replacement (Barry et al. 2006) and at post mortem examination (Ireland and Rowlands 1981). Mitral valve chordae are thick and shortened, valve edges are thickened and rolled. The aortic valve cusps are thickened throughout and some fusion of the commissures has been noted (Ireland and Rowlands 1981). Characteristic vacuolated cells deep within the valve tissue (likely valvular interstitial cells), appear to be present as well (Barry et al. 2006; Factor et al. 1978). The myocardium can be hypertrophied (Ireland and Rowlands 1981), and coronary intimal sclerosis may be present (Factor et al. 1978), even at a young age.

### Respiratory system

Alterations in respiratory function are common in patients with MPS IVA (Montaño et al. 2007). Respiratory impairment occurs due to direct involvement of respiratory tissues and as a consequence of involvement in other body systems. Thus, the etiology for respiratory impairment is multifactorial and attributable to upper and lower airway obstruction, cervical myelopathy, and chest wall restriction. Alteration in growth and development provides an additional mechanism for respiratory impairment due to the short stature and skeletal dysplasia that is frequently noted in these patients (Tomatsu et al. 2011). Patients with MPS IVA are at increased risk for complications that include recurrent infections, progressive loss of respiratory function, sleep disordered breathing, and ultimately respiratory failure (Montaño et al. 2007; Tomatsu et al. 2011; Pelley et al. 2007).

As in other MPS diseases, pulmonary involvement occurs in part due to GAG accumulation throughout the respiratory system (Peters et al. 1985; Semenza and Pyeritz 1988; Walker et al. 2003). GAG accumulation is prominent in the upper airways and tonsils leading to an increased risk for development of obstructive sleep apnea (Montaño et al. 2007). During wakefulness, collapse of the upper airway has been documented upon neck flexion (Pritzker et al. 1980). Hyperextension of the neck (the sniff position) may occur in order to maintain airway patency. GAG accumulation has also been documented in the intrathoracic airways (Semenza and Pyeritz 1988). Tracheal and bronchial wall abnormalities have been noted on post mortem examination (Walker et al. 2003). Tracheomalacia and bronchomalacia with associated airway collapse have been documented upon fiber optic bronchoscopy (Walker et al. 2003). Recognition of this complication is imperative as airway obstruction may persist after therapeutic tracheostomy due to persistent airway collapse distal to the tip of the endotracheal tube (Pelley et al. 2007). Lastly, although the incidence and magnitude of GAG accumulation in pulmonary parenchyma are unknown, if present, this complication would further compromise pulmonary function and gas exchange.

Respiratory compromise also occurs in patients with MPS IVA due to involvement of the chest wall and neuromuscular systems (Buhain et al. 1975; Sly 1980; Ashraf et al. 1991). Chest wall deformities including pectus carinatum and kyphoscoliosis can limit lung expansion and produce a restrictive impairment that manifests as a reduction in lung volume. Displacement of the diaphragm into the thoracic cavity may occur due to short stature coupled with hepatic and/or splenic enlargement, further compromising respiratory function. In addition to the previously stated chest wall abnormalities, atlantoaxial instability and spinal cord compression are common in patients with MPS IVA (Tomatsu et al. 2011) and may result in respiratory muscle weakness. Spinal cord compression can to lead to phrenic nerve dysfunction and inspiratory muscle weakness potentially impairing patient ability to maintain ventilation in the setting of reduced chest wall compliance. Furthermore, impairment of the expiratory muscle strength due to thoracic and/or lumbar involvement impairs cough and clearance of secretions, thereby predisposing patients to infections. The combined effects of these alterations are recognized mechanisms for development of respiratory failure in otherwise healthy subjects and, therefore, are likely to result in reduced ventilation rates and development of respiratory failure in patients with MPS IVA.

Respiratory function is also altered by the short stature and skeletal dysplasia characteristic of patients with MPS IVA. In other MPS disorders associated with short stature (e.g., MPS VI), patient height is the primary determinant of vital capacity (Swiedler et al. 2005). In addition, improvement in pulmonary function during enzyme replacement therapy (ERT) in other MPS disorders is frequently associated with growth and increased stature (Harmatz et al. 2010). While corresponding data in patients with MPS IVA are not available, a similar pattern is likely to occur, suggesting that measures targeted to promote growth and development may have a beneficial effect on the respiratory dysfunction characteristic of MPS IVA.

Sleep disordered breathing (SDB) is common in all MPS diseases (Semenza and Pyeritz 1988) and may precede development of overt respiratory failure during wakefulness. Ventilatory abnormalities during sleep include obstructive sleep apnea as a consequence of GAG accumulation in the upper airway and sustained hypoventilation due to the chest wall deformity and/or respiratory muscle weakness.

If chronic respiratory insufficiency and sleep disordered breathing remain unrecognized and untreated, progression to development of cor pulmonale may occur. The combined primary and secondary effects of GAG accumulation in patients with MPS IVA can result in impairment of respiratory function to a degree that can ultimately lead to respiratory failure and early demise (Pelley et al. 2007; Walker et al. 2003).

### Overall quality of life

As the MPS IVA disease process progresses, quality of life for the patient declines. Patients become progressively more dependent on care givers as deteriorating vision, hearing, oral health, respiratory and cardiac function, muscular strength, and endurance make routine daily activities increasingly difficult to accomplish. A description of the endurance and mobility challenges faced by MPS IVA patients is provided online as supplemental material (S1). Ultimately, in the absence of treatment, patient quality of life continuously decreases as the disease progresses.

## Recommended assessments

### Visual system

Annual eye examinations are recommended in MPS IVA and should include slit-lamp biomicroscopy of the cornea, measurement of intraocular pressure, assessment of refractive error, and examination of the posterior segment. Older patients may undergo electroretinography to detect the rod-cone retinal dystrophy that has been reported. If vision is reduced, evaluation with low-vision aids should be considered. When glaucoma is detected, or keratoplasty or cataract surgery is required, more frequent examinations may be necessary.

### Auditory system

Annual auditory assessments are recommended from birth (or diagnosis). Otoacoustic emissions (OAEs) can be used to ascertain outer hair cell function, however, due to the likely presence of middle ear effusions, OAEs are unlikely to be recordable, so assessment using ABR is recommended as an appropriate neonatal test. If hearing impairment is discovered, management is dependent on the severity and type of hearing loss. A conductive hearing loss for instance can be treated either with ventilation tubes if the cause is middle ear fluid, or to avoid surgery, a bone conduction hearing aid can be used, if this is fitted onto a soft band, which can be particularly good for young babies and infants.

Beyond infancy, regular audio behavioral testing should be carried out annually due to the progressive nature of the hearing impairment, both to check for any conductive elements and also because the risk of developing a sensorineural loss increases with age. From around 8 months to the age of 2.5 years, the child should be able to perform behavioral testing such as visual reinforcement audiometry (VRA), although this requires the child to be able to sit supported around the waist and have good head control. Full ear specific testing with bone conduction is possible when performing VRA at this age and is highly recommended. Once the child is 3 years of age, conventional hearing checks such as play audiometry should take place at least annually and more regularly if hearing aids have been issued.

### Digestive system

Overall digestive health should be assessed at diagnosis and then as clinically indicated. Annual imaging by ultrasound, CT, or MRI to determine liver size may be useful. The oral health component of digestive health should ideally be assessed every 6 months, but, at minimum, should be assessed at least annually. Clinicians and dentists should be aware that due to enamel defects and the common difficulties these patients experience in maintaining good oral health, MPS IVA patients have an increased risk of developing caries and may be considered as candidates for fissure sealing. It is also important to ensure patients are receiving adequate fluoride supplementation as that is the only chemical element known to be effective for caries prevention (Bromo et al. 2011). Attention to oral health is of particular importance in this patient population especially as dental caries can contribute to the risk of infective endocarditis (Franco et al. 1996), and antibiotic prophylaxis may be indicated in some cases before invasive surgery.

### Cardiovascular system

Upon establishment of diagnosis, individuals with MPS IVA should undergo baseline cardiac evaluation, including auscultation, electrocardiogram, and cardiac ultrasound to assess cardiac involvement. Although cardiac murmurs may be present, their absence does not rule out the presence of cardiac valve pathology, and noninvasive testing should still be performed. The electrocardiogram will document the presence of normal atrioventricular conduction as well as the absence of acute ischemia or previous myocardial injury. Cardiac ultrasound will provide an understanding of the underlying anatomy and function of the MPS IVA heart. The cardiac ultrasound should include the following:

1. M-mode determination of cardiac chamber dimensions and wall thicknesses and shortening fraction
2. B-mode assessment of the thickness of cardiac valves, the measurement of aortic sinus and sinotubular ridge dimensions, the left ventricular ejection fraction, and the identification of regional wall motion abnormalities, if present
3. Doppler interrogation of all cardiac valves for stenosis and/or regurgitation, of both great vessels; and finally estimation of right ventricular systolic and pulmonary artery diastolic pressures

Depending upon the initial cardiac evaluation, cardiac follow-up should be performed every 1–3 years and prior to any proposed major operative intervention(s). Should concern for potential coronary artery involvement be raised by symptoms or resting noninvasive findings, noninvasive stress imaging should be performed.

### Respiratory system

There are many methods available for respiratory assessment. Lung function and SDB should be evaluated in all patients at diagnosis and then as indicated. Children can generally perform simple respiratory function testing from the age of 5, and it is suggested that from age 8 assessment of respiratory function be performed at least annually. However, due to the width of the phenotypic spectrum of MPS IVA, identification of the most appropriate methods of assessment needs to be done on an individual patient basis.

For lung function studies age, size, and fitness are all important considerations. As patients with MPS IVA exhibit a restrictive and an obstructive element of respiratory compromise, both aspects need to be assessed. A brief discussion of assessment methods potentially appropriate for patients with MPS IVA is provided here, a more in-depth version is available online as supplemental material (S2). It should be noted that lung function tests are not diagnostic; instead they describe the pattern of a disease. Therefore, serial measurements are recommended to follow the natural progress of the disease or assess the impact of treatment.

Assessment of airflow limitation can be divided into active and passive measurements. Spirometry is the most common test utilized to assess airflow limitation. This test requires the active participation of the patient to determine the maximum volume of air that can be exhaled in the first second of a forced expiratory maneuver starting at full lung inflation (forced expiratory volume in 1 s, FEV_1_). However, due to the need to assess very young patients and the common lack of endurance in older patients, passive measurements may be appropriate. One such measurement is impulse oscillation (IOS), where airway resistance is assessed utilizing a speaker to impose external oscillating pressure and airflow impulses over the subject’s tidal breathing. A second type of passive measurement, multiple-breath inert gas washout (MBW), has the potential to be used across all age ranges (including infants). Airway disease is identified using an index of the uniformity of airflow distribution throughout the airways. Since MBW measurements vary minimally with age, MBW should be particularly useful for following children longitudinally (Robinson et al. 2009) as would be desirable in following disease progression in a patient with MPS IVA.

Additional measurements should include assessment of lung size. Spirometry is a readily available technique utilized to measure the vital capacity (VC; the maximum amount of air that can be inhaled/exhaled from full inflation to maximum deflation) providing an indication of the size of the lungs. The “gold standard” assessment of lung restriction is by measurement of total lung capacity (TLC). This can be assessed by a variety of techniques either physiological, including whole body plethysmography, gas (typically helium) dilution, and nitrogen washout, or by radiographic means [conventional chest radiographs or computerized tomography (CT)]. In an ideal world both plethysmography and gas dilution/nitrogen washout tests should be performed, however this may be limited by the availability of equipment and local expertise. In patients with limited ability to co-operate (such as children 2–5 years of age), radiographic techniques may offer an alternative method of assessment (Clausen 1997).

Sleep studies should be performed in addition to lung function assessments. Performance of a formal sleep study is recommended at diagnosis and should be repeated whenever SDB is suspected. Symptoms suggestive of SDB, such as apneas, gasping respirations, snoring, difficult wakening, daytime somnolence, and restless sleep, should be assessed as part of the clinical evaluation of all patients at least annually. Overnight sleep studies can be used to both diagnose the type and severity of SDB and to evaluate nocturnal ventilatory treatments for the underlying respiratory disorder.

All patients should be asked about symptoms of respiratory insufficiency such as dyspnea; however, symptoms may be minimally perceived when exercise capacity is limited secondary to reduced endurance. Objective assessment of CO_2_ retention is best accomplished by analysis of arterial blood gases, however, this test may not be readily available in some outpatient settings. Alternatively, capnography may be used as a surrogate measure, or elevation of serum bicarbonate concentration in venous blood can be used as a screening test for chronic hypoventilation; an elevated serum bicarbonate concentration (>27 mEq/L) may be a marker of renal compensation for chronic CO_2_ retention.

Lastly, spiral CT of the chest can be useful in detecting tracheomalacia and should be considered at least before major surgery to uncover upper airways abnormalities (Shih et al. 2002).

### Overall quality of life

To track and manage quality of life, patients with MPS IVA should see a specialist physiotherapist annually to carry out evaluation of impairment, including determination of range of motion, muscle strength, and resulting activity and participation limitations.

Annual endurance assessments are recommended as a method of following patient mobility and potential for independence. Because endurance measurements allow for the clinical evaluation of multiple systems simultaneously, they are an effective method of assessing overall disease progression (McDonald et al. 2010). Reductions in endurance alter patients’ functional capacity, which can lead to decreased independence and therefore impact on their quality of life.

Patients should also undergo evaluation by a physiotherapist and endurance assessments prior to and after surgeries to enable clinicians to establish a baseline and track post-operative progress.

Recommended endurance measurements for patients with MPS IVA include the six-minute walk test (6MWT) as described by the American Thoracic Society (ATS 2002) and the three-minute stair climb (3MSC) as described for patients with MPS VI (Harmatz et al. 2005). A description of these tests as well as a discussion of complications and limitations is available online as supplemental material (S3).

## Interventions and outcomes

### Visual system

Conservative treatment for reduced vision and photosensitivity due to opacification of the cornea in MPS IVA includes filtering or photochromic spectacles and a brimmed hat, visor, or cap (Northover et al. 1996). When corneal clouding is marked, keratoplasty may be required. Important considerations include other possible causes of reduced vision (Käsmann-Kellner et al. 1999), other sensory disabilities (e.g., hearing loss), neurodevelopmental status, and availability of an anaesthesia team skilled in working with individuals with MPS (Belani et al. 1993; Geetha et al. 2010). Recurrence of corneal opacity after 10 months was reported in a 12-year-old boy with MPS IVA (Käsmann-Kellner et al. 1999). Others have reported reopacification of the transplanted cornea in MPS IVA as well (Maumenee 1978; Iwamato et al. 1990). However, clear corneal grafts have been reported 2 years (Leslie et al. 2005) and 6 years (Couprie et al. 2010) after keratoplasty. Reopacification of the corneal graft may occur more frequently in MPS disorders in which keratan sulfate accumulates, compared with MPS disorders in which heparan sulfate or dermatan sulfate accumulates (Käsmann-Kellner et al. 1999). Despite reopacification of the cornea, keratoplasty may provide substantial improvement in daily function for a variable period of time in patients with MPS IVA. Interestingly, corneal clouding has also reportedly improved following successful bone marrow transplantation (Desai et al. 1983).

When visually significant cataracts occur in patients with MPS IVA (typically later in life), cataract surgery with placement of an intraocular implant can restore vision if other irreparable causes of vision loss are absent (Couprie et al. 2010). Glaucoma rarely occurs in patients with MPS IVA and may initially be managed medically. With progressive optic nerve cupping and/or visual field loss, filtration surgery may be required. When reduced vision cannot be further improved with refractive correction, patients may benefit from low-vision aids, in addition to high contrast and enlarged print material. However, there is no current treatment to specifically ameliorate the optic atrophy and retinal dystrophy that have occasionally been reported in this disease. With periodic reassessment, the patient’s quality of life can be maximized by treatment of the ocular abnormality (e.g., keratoplasty or glaucoma treatment) or providing options to enhance the use of residual vision.

### Auditory system

By the first decade of life, most patients with MPS IVA may be expected to have either a sensorineural hearing loss or a mixed hearing loss, which is a combination of sensorineural and conductive hearing loss (Riedner and Levin 1977).

Ventilation tubes can be offered to treat conductive hearing loss from retained middle ear fluid. These are placed under general anaesthetic, where a small incision behind the tympanic membrane can be made (myringotomy) to drain the accumulated fluid. A small ventilation tube often called a grommet may then be inserted, which allows air to enter. Grommets will eventually fall out, and once they have fallen out the tympanic membrane will heal, and the fluid can re-occur. T-tubes are a longer lasting type of grommet, and in view of the anaesthetic risks for individuals with MPS IVA, and the risk of the reoccurrence of the middle ear fluid, may be preferable to use on the first occasion.

Bone conduction hearing aids are a nonsurgical option for the first few years of life and can be worn either on a headband or a soft band. These are preferable to air conduction hearing aids if the hearing loss fluctuates, which is often the case with conductive hearing losses caused by middle ear fluid.

As there is a progressive sensorineural element to the hearing loss in MPS IVA, bone conduction hearing aids may cease to be the most appropriate forms of amplification. Post-aural hearing aids may be needed to deliver the gain required for the patient to access the speech frequencies and ideally will work best if the fluctuating conductive element of the hearing loss is reduced by use of ventilation tubes.

### Digestive system

Hernias can be repaired surgically, and due to poor connective tissue, special techniques may be needed to prevent recurrence. Approximately 6% of the patients in the International Morquio A Registry reported undergoing herniorrhaphy; the mean age was 8 years at the time of surgery (Montaño et al. 2007). As with any surgical procedure in this patient population, anaesthesia should be performed with caution, and to prevent potential complications, anaesthesiologists should have experience working with MPS patients due to the difficult airway, short stature, restricted head movement, and other features of this multisystem disease.

Regarding oral health, parents or care givers should be given advice on maintaining their child’s oral hygiene. Regular preventative maintenance is highly recommended to avoid the need for interventions requiring general anaesthesia.

### Cardiovascular system

Defining the medical management for the valvulopathies that may occur in MPS IVA is beyond the scope of this review. Existing guidelines for the medical management of cardiac valve disease have been constructed for common valves disorders found in normal adults, such as aortic stenosis from calcification of a bicuspid aortic valve (Bonow et al. 2006, 2008), but not for the MPS diseases specifically. These guidelines are a reasonable starting point but do not address all of the other organ systems (pulmonary, skeletal) that may influence the outcome for individuals with MPS IVA.

Similarly, indications for cardiac valve replacement in MPS IVA have not been defined. Noncardiac factors, such as cervical instability and respiratory function, should be factored into the timing of valve replacement. Familiarity with the airway, respiratory, and CNS issues of MPS patients is important in the anaesthetic and perioperative management of patients with MPS IVA. Intraoperative monitoring of ongoing peripheral nervous system function should be part of the standard intraoperative protocol. Successful aortic valve replacement has been reported in adults with MPS IVA (Pagel and Almassi 2009; Nicolini et al. 2008). The long term failure of the Ross procedure (in which the patient’s own pulmonary valve is used to replace the stenotic or regurgitant aortic valve) has been reported in MPS IVB (Barry et al. 2006). Finally, bone marrow transplantation has been performed in two children with MPS IV (subtype A or B not specified): a 5 year old (Gatzoulis et al. 1995) who demonstrated no improvement in cardiac status and an 8 year old (Kato et al. 1986) in whom cardiac follow-up was not provided.

### Respiratory system

Multiple interventions are required to maintain optimal functional status, and patients may benefit from involvement of a pulmonologist, preferably with experience managing patients with MPS disorders. Therapy should be targeted based on the underlying abnormalities and pulmonary status of each individual. All patients should receive regular vaccinations, including pneumococcus and influenza vaccinations. Treatment of respiratory tract infections should be early and aggressive. Impairment in secretion clearance due to chest wall abnormalities and weakened cough can be addressed by both manual and mechanical techniques. Inhaled bronchodilators can be used in conjunction with these airway clearance techniques. Bronchodilators and corticosteroids (inhaled and/or oral) may be useful if concurrent asthma is present.

Abnormal sleep study results indicate intervention is needed and upper airways should be evaluated. Removal of tonsils and adenoids, if enlarged, could help with upper airway obstruction. While removal of abnormal deposits is sometimes useful, it should be left to those with experience in this procedure as upper airway scar tissue could compromise the already small airway (Yeung et al. 2009). Upper airway obstruction and progressive airway collapse can also be managed by respiratory support and CPAP (continuous positive airways pressure), which acts as a dynamic airway stent. For patients with nocturnal hypoventilation, the use of noninvasive ventilator support systems (e.g., bilevel positive airway pressure, BiPAP) can dramatically improve quality of life. Patients with abnormal posturing (the sniff position) may require specialized attention to ensure that the mask used is comfortable and adequate. Supplemental oxygen can be used if these methods do not fully correct the hypoxia, but careful titration of the rate of oxygen administration and monitoring of arterial carbon dioxide levels are required to prevent oxygen-induced hypercapnia. Tracheostomy is sometimes needed especially if tracheomalacia is present but is a particular problem in this cohort due to persistent airway collapse distal to the tip of the endotracheal tube (Pelley et al. 2007) and the patients’ preference for sleeping on their stomachs. Sadly, uncontrolled respiratory failure is frequently the event leading to death in these patients.

### Overall quality of life

The current options for treating MPS IVA in a multisystemic manner to address overall quality of life are limited. Although bone marrow transplant is an option, the procedure has high rates of morbidity and mortality due to infection, graft-versus-host disease, and other complications (Tomatsu et al. 2011). ERT has been used successfully in other MPS disorders (Wraith et al. 2004; Muenzer et al. 2006; Harmatz et al. 2006), but it is not yet available for patients with MPS IVA. However, ERT clinical trials for MPS IVA are currently underway (http://clinicaltrials.gov), which may lead to ERT becoming a treatment option in the future.

Currently, in the absence of an effective and safe systemic treatment option, patients require extensive management and regular intervention to maximize their quality of life. Although their quality of life will continually decline over their lifetime as the disease progresses, steps can be taken along the way to keep patients active, pain free, and independent for as long as possible. A brief discussion of some of these steps is available online as supplemental material (S4).

## Conclusions

Non-skeletal manifestations of MPS IVA, including the visual, auditory, digestive, cardiovascular, and respiratory system abnormalities, have a significant impact on patient quality of life. Regular assessments of these systems are recommended for all patients with MPS IVA (Table 1) to facilitate timely intervention and maximize quality of life potential.

**Table 1:** Recommended assessments for non-skeletal aspects of MPS IVA

	At diagnosis	Annually	Every 1–3 years	Prior to major operative interventions	Other
**Visual system**					
Slit-lamp biomicroscopy of cornea	●	●			More frequent examinations may be necessary if keratoplasty or cataract surgery is required
Measurement of intraocular pressure	●	●			More frequent examinations may be necessary if glaucoma is detected
Assessment of refractive error^a^	●	●			
Examination of posterior segment^b^	●	●			
Electroretinography under scotopic and photopic conditions					At onset of symptoms (night blindness or constriction of visual field) or signs (arteriolar attenuation, retinal pigment migration), with reassessment every 5 years
**Auditory system**					
Conventional hearing checks (≥3 years old)	●	●			
OAE					At diagnosis in neonates
VRA					At diagnosis and annually for patients between 8 months and 3 years old
**Digestive system**					
Clinical evaluation of digestive health	●				As clinically indicated
Imaging by ultrasound, CT, or MRI to determine liver size		●			
Evaluation of oral health by a dentist		●			Ensure adequate fluoride supplementation and consider fissure sealing of dentition
**Cardiovascular system**					
Auscultation	●		●	●	
Electrocardiogram	●		●	●	
Cardiac ultrasound	●		●	●	
Noninvasive stress imaging					As clinically indicated
**Respiratory system**					
Spirometry to assess VC and FEV_1_	●	●			
IOS					When spirometry measurements are not feasible
MBW					When following disease progression from an early age is desired
TLC determined by gas dilution or washout methods (≥8 years old)	●	●			
TLC determined by plethysmography (≥8 years old)					If equipment and expertise are available
TLC determined by radiographic techniques					In place of other TLC determination methods in patients with limited ability to co-operate
Clinical evaluation of symptoms suggestive of SDB	●	●			
Overnight sleep studies	●				As clinically indicated
Analysis of arterial blood gases	●	●			As clinically indicated
Capnography					If an analysis of arterial blood gases is not available
Serum bicarbonate concentration in venous blood					If an analysis of arterial blood gases and capnography are not available
Spiral CT of the chest				●	
**Overall quality of life**					
Evaluation of impairment by a physiotherapist	●	●			Prior to and after surgical procedures to track patient progress and as clinically indicated
6MWT (if developmentally and physically able)	●	●			Prior to and after surgical procedures to track patient progress and as clinically indicated
3MSC (if developmentally and physically able)	●	●			Prior to and after surgical procedures to track patient progress and as clinically indicated

*OAE* Otoacoustic emissions, *VRA* visual reinforcement audiometry, *VC* vital capacity, *FEV*_*1*_ forced expiratory volume in 1 s, *IOS* impulse oscillation, *MBW* multiple-breath inert gas washout, *TLC* total lung capacity, *SDB* sleep disordered breathing, *6MWT* six-minute walk test, *3MSC* three-minute stair climb

^a^Correction of refractive error may include filtering spectacle lenses if photosensitivity is present. Bifocals or reading glasses may be needed if vision is reduced

^ b^Evaluate optic nerve (edema, atrophy, cupping), vessels (arteriolar attenuation), and retina (pigmentary change)

Ophthalmologic abnormalities develop gradually, but if regularly assessed, can be identified and usually corrected to prevent impairment. The progressive and multifactorial decline of auditory function is also likely to require intervention. Maintaining a patient’s ability to see and hear by utilizing visual and auditory system assessments and interventions can help maintain patient functionality and independence. Treating any dental disease early and performing preventative dental maintenance are important in avoiding oral interventions requiring general anaesthesia and maintaining overall oral health and patient quality of life.

Assessment of cardiovascular and respiratory systems is of paramount importance. Cardiovascular and respiratory interventions can be life-saving in MPS IVA. Familiarity with the cardiovascular and respiratory issues in MPS IVA and appropriate baseline measurements are important during operations and other medical procedures requiring sedation. Respiratory complications in particular are often responsible for patient losses.

The non-skeletal manifestations of MPS IVA are responsible for significant morbidity and mortality in the MPS IVA population. However, regular assessments and timely interventions can substantially improve patient outcomes.

## Electronic supplementary material

Below is the link to the electronic supplementary material.

ESM 1 (DOCX 24.3 kb)
