# Supplementary material for: Clinical overview and treatment options for non-skeletal manifestations of mucopolysaccharidosis type IVA
Source: J Inherit Metab Dis. 2012 Feb 23;36(2):309–22. doi: 10.1007/s10545-012-9459-0 (PMC3590399; doi:10.1007/s10545-012-9459-0)
Supplement: Supplementary file 1 — (DOCX 24.3 kb) [file 10545_2012_9459_MOESM1_ESM.docx]

**SUPPLEMENTAL INFORMATION**

**S1. Endurance and Mobility Challenges**

Reduced endurance in particular has a large impact on most patients with MPS IVA (Montaño et al. 2007). Previously mentioned respiratory and cardiovascular impairments play a significant role, alongside classic musculoskeletal abnormalities, in reducing endurance. Musculoskeletal abnormalities range in severity and, therefore, their impact on endurance can vary. Significant endurance-impairing issues caused by the musculoskeletal system include gait abnormalities, joint pain, and muscle weakness. The typical posture and gait pattern adopted by patients with MPS IVA is one with head cocked back in the sniff position, hips and knees flexed, a tendency to valgus posturing at the knees, and pronation of the feet. Arm swinging, often adopted to aid with propulsion as muscle strength declines, can be vigorous and often leads to a waddling gait pattern. The upward gaze of the sniff position leads to further difficulty maneuvering. Additionally, pain in the lumbar spine, hips, knees, and ankles has been reported by patients with MPS IVA (Montaño et al. 2007). Orthopedic procedures are common (Montaño et al. 2007) and can be beneficial in improving joint alignment, reducing pain, and potentially maintaining patient mobility. However, post surgical rehabilitation and endurance conditioning have been found to be particularly difficult in some patients with MPS IVA due to muscle weakness. As muscle weakness increases with disease progression, endurance is reduced and dependence on care givers increases.

Progression of musculoskeletal, respiratory, and cardiovascular impairments leads to deterioration of functionality which may result in the patient becoming wheelchair bound and possibly requiring an electric wheelchair for independent mobility. Data from an international registry indicate that over 30% of patients with MPS IVA use wheelchairs (Montaño et al. 2007).

Reductions in mobility and endurance lead to decreased quality of life and motivation. Engagement in the activities of daily life becomes more onerous; for example, in children walking around school may become too difficult and lead to walking during play time only, or a self-propelled wheelchair may be exchanged for an electric wheelchair. Walking up stairs may become difficult due to a combination of muscle weakness and decreased endurance. Use of railings and banisters is typically required, although, due to short stature, poor shoulder range of motion, and weakness in hands and wrists, using normal height railings can be very difficult. Young patients with MPS IVA often resort to using hands and knees to ascend stairs or are carried by their parents or care givers.

**S2. Lung Function Assessment**

Assessment of airflow limitation can be divided into active and passive measurements. Active measurements include the Maximal Flow Volume Loops (MFVL) and Maximal Voluntary Ventilation (MVV) whilst passive measurements include Impulse Oscillation (IOS) and Multiple-Breath Inert Gas Washout (MBW).

Spirometry is one of the most common respiratory assessment tools and is useful in detecting mechanical abnormalities of the airways. It assesses either volume or flow changes with respect to time as the subject performs a maximal expiratory and inspiratory manoeuvre. MFVL uses this tool; however, it is a physically demanding test that requires a certain level of mental co-operation and physical co-ordination from the subject and a high level of interaction and motivation from the tester. While incentive spirometry has progressed significantly over recent years it is still vital that the tester has extensive experience of performing these tests in children to ensure proper execution of the manoeuvre (Beydon et al. 2007).

The MVV is the maximal volume of air that a subject can breathe using repetitive maximal respiratory efforts over a given time period, typically between 12–15 seconds. The MVV is reduced in airflow obstruction but is relatively well preserved in restrictive lung disease. It is however an intensely demanding and exhausting test and in most cases yields little additional data in comparison to measurements of a single maximal expiration (Comroe et al. 1962).

In contrast to active measurements made using maximal effort, it is possible to assess airway abnormalities using passive measurements during tidal breathing. Due to the need to assess very young patients and the common lack of endurance in older patients, passive measurements may be the most appropriate measurements for many patients with MPS IVA. One such measurement is IOS where a speaker is used to impose external oscillating impulses over the subject’s tidal breathing. Mathematical analysis of the flow signal generated by the impulses and the resultant pressure response gives the respiratory impedance of the pulmonary-thoracic system. This in turn can be differentiated between the proximal and distal components of the respiratory tract. Assessment of the values obtained and the curves generated should allow easy differentiation between obstructive and restrictive elements of respiratory compromise (Hellinckx et al. 2001).

Another fairly recent test that may prove suitable for patients with MPS IVA is MBW which is reported to be an extremely sensitive method of detecting early lung disease including that in the lung periphery (Aurora et al. 2005; Robinson et al. 2009). This test measures the Lung Clearance Index (LCI) which is a measure of the total expired volume required to washout an inert tracer gas from the lungs which is then divided by lung volume to correct for size. Because MBW is performed during tidal breathing, it has the potential to be used across all age ranges (including infants) and since the LCI appears to vary minimally with age, the MBW should be particularly useful for following children longitudinally (Robinson et al. 2009) as would be desirable in following disease progression in a patient with MPS IVA.

Whilst measurement of the vital capacity (VC) by spirometry gives an indication of the size of the lungs the ‘gold standard’ assessment of lung restriction is by measurement of Total Lung Capacity (TLC). This can be assessed by a variety of techniques either physiological, including whole body plethysmography, gas (typically helium) dilution, and nitrogen washout, or by radiographic means (conventional chest radiographs or computerized tomography (CT)). Each of the physiological techniques actually measures the subject’s Functional Residual Capacity (FRC) and then by the addition of a VC manoeuvre allows the calculation of the TLC and Residual Volume (RV). Whilst it is normally recommended to perform this VC manoeuvre as a maximal expiration followed by a maximal inspiration, in the MPS IVA patient group it is generally better to perform a maximal inspiration to calculate the TLC. Subtraction of the maximal VC from TLC will then give the RV.

Plethysmography measures all of the gas contained within the thoracic cage (including abdominal) even if trapped and as such gives slightly higher readings than either the gas dilution or washout methods. In a normal subject these differences should be minimal (<500 mL), however in subjects with significant airflow limitation the differences will become more significant and reflect the size of poorly ventilated areas. In an ideal world both plethysmography and gas dilution/nitrogen washout tests should be performed, however this may be limited by the availability of equipment and local expertise. A detailed technical review of the methods for performing these tests has been published in the European Respiratory Journal (Miller et al. 2005; 2005)

In patients with limited ability to co-operate (such as children 2 to 5 years of age) radiographic techniques may offer an alternative method of assessment (Clausen 1997). However, it is not possible to do a direct comparison between these methods and physiological ones.

One of the most difficult questions posed by the MPS IVA patient population is which predicted values should be used. Predictive equations are used to express pulmonary function in relation to that which would be expected for healthy individuals of similar age, sex, body size, and ethnic group. Without knowledge of the ‘normal’ range of results it is impossible to distinguish the effects of disease and treatment from normal variability inherent in any lung function test. Unfortunately, whilst there is ample data available for normal adults, there is a distinct lack of normative data for children and those of short stature. Whilst efforts are being made to fill this gap (Stanojevic et al. 2009) much thought needs to be given to which predicted values should be used for patients severely affected by MPS IVA since their height (which is the main component in determining lung function) rarely exceeds 105 cm. A reliable baseline needs to be established for this patient population

**S3. Endurance Assessments**

The six minute walk test (6MWT) is a straightforward test that can be carried out using a 30 m hallway. The patient is tasked to walk as quickly as possible for 6 minutes between 2 cones marking the 30 m course. It is a self-paced exercise test which assesses the patient’s submaximal functional capacity. The patient is allowed to take rests if needed but is required to continue when able. This test does not require expensive or specialized equipment or training to carry out. This is also true of the three minute stair climb (3MSC) which tasks the patient with ascending as many stairs as possible in 3 minutes, using railings and resting as necessary. Blood oxygen saturation and heart rate measurements are taken immediately prior to the test, upon test completion, and 2 minutes after test completion.

Several complicating factors should be taken into consideration when performing these tests and interpreting results. Blood oxygen saturation and heart rate measurements can be difficult to obtain at times due to small nail beds and cold extremities. Poor peripheral circulation and decreased muscle pump due to muscle weakness may be contributing factors to these testing complications common among patients with MPS IVA. Environmental issues are also a factor when assessing functional capacity. Corridors that are busy and crowded, overly hot in the summer if not air-conditioned, or particularly cold in the winter may influence individual patient performance during assessment. Positive motivation due to parental encouragement or competition between siblings or friends can also be a factor.

When these tests are repeated more often than annually, additional factors should be considered in the interpretation of results. For example, if repeat measurements are desired and endurance is assessed on contiguous days, compliance and motivational issues, both positive and negative, can influence results. Some patients may experience extreme fatigue or pain following initial testing and the duplicate testing may show a poorer result as a consequence of soreness or fear of pain.

**S4. Maintaining Quality of Life**

For children with MPS IVA communication among school physiotherapists, health professionals, and school staff regarding inclusion in activities at school is important. Specialist advice and training sessions with schools are essential regarding specialized equipment, light balls, walking aids, etc to enable children with MPS IVA to participate as fully as possible in everyday life. Advice regarding level of activity is particularly important when mobility is decreasing and the option of wheelchair mobility is being raised. Balance between independence and maintaining mobility needs to the considered to ensure the patient’s quality of life is not compromised more than necessary. Adaptations can also be made to the home environment to facilitate maximal independence. Additionally, orthotic intervention is considered useful for provision of footwear and in some cases, spinal jackets, insoles, collars, etc are provided after surgery or for management of specific issues.

Patients with MPS IVA undergo a variety of orthopedic and spinal surgeries throughout their lives (Montaño et al. 2007). Post operative rehabilitation needs to be timely and aimed at restoring and building upon the level of preoperative functioning. While experts acknowledge timing of interventions is crucial, a consensus has not yet been reached. Due to the muscle weakness and fatigue, therapy should be paced and graded. Anecdotally, muscle strengthening following spinal and knee surgery in a number of children with MPS IVA has proved beneficial in improving post morbid status.

**Supplemental Table 1.** Incidence of cardiac ultrasound findings in MPS IVA and undefined MPS IV in studies with Doppler.

| **MPS Type** | **Study** | **No. of Patients** | **Average Age in Years**  **(range)** | **Aortic/Mitral Valve** | | |
| --- | --- | --- | --- | --- | --- | --- |
|  |  |  |  | **Thickening** | **Regurgitation** | **Stenosis** |
| IVA | John et al. 1990 | 10 | 12.5  (3–40) | 3/3 | 3/5 | 0/1 |
| IV | Wippermann et al. 1995 | 12 | 12.5  (2–47) | 5/11 | 1/4 | 3/2 |
| IV | Dangel 1998 | 5 | 12.1  (7–25) | 3/5 | ND/ND | ND/ND |
| IV | Mohan et al. 2002 | 16 | ND | 4/1 | 4/2 | 1/0 |
| IV | Fesslova et al. 2009 | 10 | 7.2  (0.3–35.7) | ND/ND | 2/3 | 0/0 |
| IV | Lael et al. 2010 | 7 | 9.5 | 5/2 | 1/3 | 0/ND |
| IV | Total from studies not differentiating MPS IVA and B (#2–6) | 46 | ND | 17/19 | 8/12 | 4/2 |

ND = no data available

**Supplemental Material References**

Aurora P, Bush A, Gustafsson P et al. (2005) Multiple-breath washout as a marker of lung disease in preschool children with cystic fibrosis. Am J Respir Crit Care Med 171: 249–256

Beydon N, Davis SD, Lombardi E et al. (2007) An official American Thoracic Society/European Respiratory Society statement: pulmonary function testing in preschool children. Am J Respir Crit Care Med 175:1304–1345

Clausen JL (1997) Measurement of absolute lung volumes by imaging techniques. Eur Respir J 10: 2427–2431

Comroe JH Jr, Forster RE, Dubois AB, Briscoe WA, Carlsen E (1962) The Lung: Clinical Physiology and Pulmonary Function Tests-Second Edition. Chicago: Year Book Medical Publishing, Chapter 7

Dangel JH (1998) Cardiovascular changes in children with mucopolysaccharide storage diseases and related disorders – clinical and echocardiographic findings in 64 patients. Eur J Pediatr 157:534–538

Fesslová V, Corti P, Sersale G et al. (2009) The natural course and the impact of therapies of cardiac involvement in the mucopolysaccharidoses. Cardiol Young 19:170–178

Hellinckx J, Cauberghs M, De Boeck K, Demedts M (2001) Evaluation of impulse oscillation system: comparison with forced oscillation technique and body plethysmography. Eur Respir J 18:564–570

John RM, Hunter D, Swanton RH (1990) Echocardiographic abnormalities in type IV mucopolysaccharidosis. Arch Dis Child 65:746–749

Lael GN, de Paula AC, Leone C, Kim CA (2010) Echocardiographic study of paediatric patients with mucopolysaccharidosis. Cardiol Young 20:254–261

Miller MR, Crapo R, Hankinson J et al. (2005) General considerations for lung function testing. Eur Respir J 26:153–161

Miller MR, Hankinson J, Brusasco V et al. (2005) Standardisation of spirometry. Eur Respir J 26:319–338

Mohan UR, Hay AA, Cleary MA, Wraith JE, Patel RG (2002) Cardiovascular changes in children with mucopolysaccharide disorders. Acta Paediatr 91:799–804

Montaño AM, Tomatsu S, Gottesman GS, Smith M, Orii T (2007) International Morquio A Registry: clinical manifestation and natural course of Morquio A disease. J Inherit Metab Dis 30: 165–174

Robinson PD, Goldman MD, Gustafsson PM (2009) Inert gas washout: theoretical background and clinical utility in respiratory disease. Respiration 78:339–355

Stanojevic S, Wade A, Cole TJ et al. (2009) Spirometry centile charts for young Caucasian children: the Asthma UK Collaborative Initiative. Am J Respir Crit Care Med 180:547–552

Wippermann CF, Beck M, Schranz D, Huth R, Michel-Behnke I, Jungst BK (1995) Mitral and aortic regurgitation in 84 patients with mucopolysaccharidoses. Eur J Pediatr 154: 98–101
